# Are Conditioned Pain Modulation and Pain Sensitivity Risk Factors for the Development of Functional Somatic Disorder? A Longitudinal Population‐Based Study

**DOI:** 10.1002/ejp.70326

**Published:** 2026-07-04

**Authors:** Lise Kirstine Gormsen, Tina Birgitte Wisbech Carstensen, Sine Skovbjerg, Thomas Meinertz Dantoft, Torben Jørgensen, Marie Weinreich Petersen

**Affiliations:** ^1^ Department of Functional Disorders Aarhus University Hospital Aarhus Denmark; ^2^ Department of Clinical Medicine Aarhus University Aarhus Denmark; ^3^ Center for Clinical Research and Prevention Capital Region and University of Copenhagen Copenhagen Denmark; ^4^ Department of Public Health, Faculty of Health and Medical Science University of Copenhagen Copenhagen Denmark

**Keywords:** chronic pain, cold pressor test, conditioned pain modulation, epidemiology, functional somatic disorder, general population, pressure pain thresholds

## Abstract

**Background:**

Disrupted pain regulation has been suggested as an important component of functional somatic disorder (FSD), but evidence from large population‐based studies remains limited. This study examined whether altered pain regulation, defined as lower pressure pain threshold (PPT) and reduced conditioned pain modulation (CPM), constitutes a risk factor for developing FSD over a 5‐year period in a large population‐based cohort.

**Methods:**

PPT was recorded at baseline. During cold pressor stimulation of the hand, PPT was reassessed and the difference from baseline defined the CPM effect. Participants were randomly selected from the adult Danish population (*n* = 2198) of whom 1269 (57.7%) participated in the 5‐year follow‐up. Incident FSD cases were identified using validated self‐reported symptom questionnaires. Associations between pain measures and incident FSD were examined using logistic regression analyses.

**Results:**

Overall, no significant associations were observed between baseline PPT (OR = 0.92, 95% CI: 0.83–1.01) or CPM (OR = 0.96, 95% CI: 0.90–1.03) and the development of FSD during the 5‐year follow‐up period. Similarly, changes in PPT from baseline to follow‐up were not associated with incident FSD (OR = 0.91, 95% CI: 0.83–1.00).

**Conclusions:**

The findings do not support that altered pain regulation is a risk factor for the development of FSD in the general population. However, uncertainty persists due to possible outcome misclassification, a response rate of 57.7%, low event numbers and measurement limitations.

**Significance Statement:**

This large population‐based longitudinal study challenges prevailing assumptions by showing that experimentally assessed pain sensitivity and conditioned pain modulation do not predict the development of functional somatic disorder (FSD). Contrary to findings from clinical samples, altered pain regulation was not a significant risk factor over 5 years. These findings question the causal role of central pain mechanisms in FSD onset and call for more research to clarify the underlying mechanisms of FSD.

## 1 Introduction

Functional somatic disorder (FSD) is characterised by persistent physical symptoms that cannot be better explained by other somatic or psychiatric conditions (Burton et al. 2020). It includes speciality‐specific syndromes including fibromyalgia (FM), irritable bowel syndrome (IBS) and chronic fatigue syndrome (CFS). A phenotype of multisystem FSD or multi‐organ bodily distress syndrome has been identified for patients with symptoms from multiple organ systems (Fink and Schröder 2010; Petersen, Schroder, et al. 2019).

Pain is a frequent symptom that is included in the delimitation of most definitions of FSD, which has made the study of pain an obvious target in the pursuit of a better understanding of the pathophysiological mechanisms triggering these conditions (Bourke et al. 2015; den Boer et al. 2019).

Pain thresholds to stimuli such as heat or pressure are commonly used to assess pain sensitivity (Arendt‐Nielsen 2015; Fischer 1987; Woolf 2011). Studies generally show hypersensitivity and lowered pain thresholds in FSD, although most have focused on selected patient populations, primarily those meeting the criteria for FM (Desmeules et al. 2003; Gormsen et al. 2012; Jespersen et al. 2007; Petzke et al. 2003), IBS (Jarrett et al. 2016; Piché et al. 2010; Stabell et al. 2013; Zhou et al. 2010) and CFS (Meeus, Nijs, et al. 2010; Meeus, Roussel, et al. 2010; Nijs et al. 2012).

The descending pain modulatory system can be assessed using the conditioned pain modulation (CPM) paradigm (Arendt‐Nielsen 2015; Fernandes et al. 2019; Gerhardt et al. 2017; Gil‐Ugidos et al. 2024; Gormsen et al. 2012; O'Brien et al. 2018; Petersen, McPhee, et al. 2019; Yarnitsky et al. 2015). CPM involves applying a conditioning pain stimulus to one site while evaluating pain sensitivity at another (Kennedy et al. 2016; Yarnitsky et al. 2010). CPM has been examined in FSD, mainly FM (Gil‐Ugidos et al. 2024; Lannersten and Kosek 2010; O'Brien et al. 2018; Potvin and Marchand 2016) and IBS (Albusoda et al. 2018; Jarrett et al. 2016).

Previous studies have shown hypersensitivity, lower pressure pain thresholds (PTT) and reduced CPM across various FSD definitions. However, most studies rely on small, homogenous samples, often using varying methodologies and including only female participants, which limits generalisability. In contrast, our previous large population‐based baseline study assessing PPT and CPM in FSD established by using self‐reported symptom questionnaires (Petersen et al. 2021) did not demonstrate these associations. Consequently, additional research in this area is needed. Moreover, studies on whether pain thresholds can predict the development of pain conditions do not exist when it comes to predicting FSD in population‐based samples.

This study aims to explore if altered pain regulation is a risk factor for the development of FSD over a 5‐year follow‐up period in a population‐based sample.

Hypotheses were: (1) low PPT and CPM at baseline were individual risk factors for developing FSD, (2) a small increase (below 20%) in PPT after a cold stimulus for the CPM was a risk factor for developing FSD and (3) a decrease in PPT from baseline to follow‐up was a risk factor for developing FSD.

## 2 Methods

### 2.1 Study Participants

Data from the Danish Study of Functional Disorders (DanFunD) (Dantoft et al. 2017). Baseline data (*n* = 7493) were collected between 2012 and 2015; 53.9% were women in the age range 18–72 years. Data for the 5‐year follow‐up cohort (*n* = 4288) were gathered from 2018 to 2020; 53.6% were women in the age range 24–77 years (Dantoft et al. 2017; Jørgensen et al. 2022). Participants for the baseline investigation were randomly selected from the Danish Civil Registration System. Exclusion criteria included being born outside Denmark, not holding Danish citizenship and pregnancy.

**FIGURE 1:**
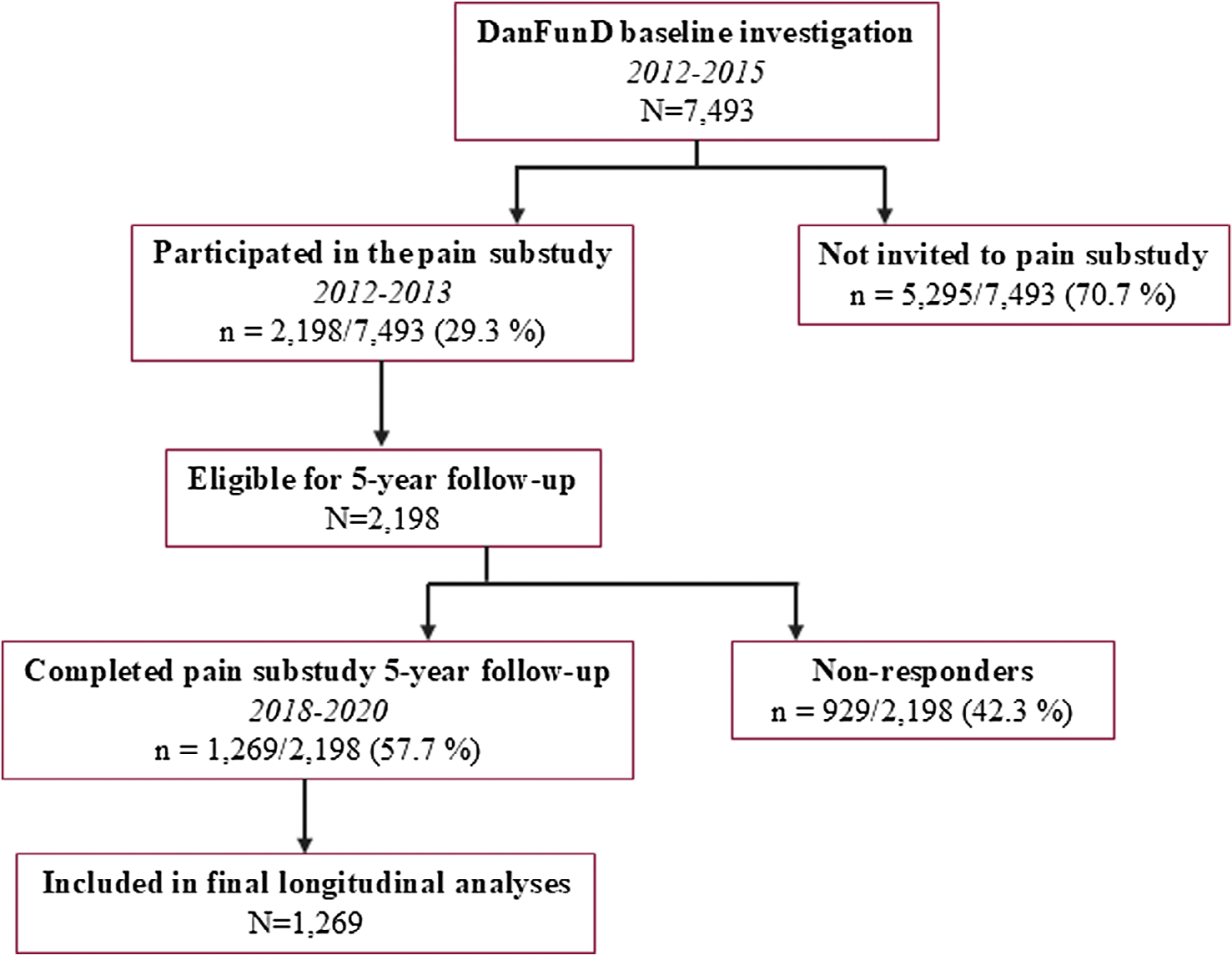
Flowchart of participant inclusion from the DanFunD baseline investigation through participation in the pain substudy, 5‐year follow‐up and final longitudinal analytical sample.

The pain substudy commenced in November 2012, with pain assessments concluded at the end of 2013 (Figure 1). A total of 2198 consecutively recruited persons from the baseline investigation took part in the measurements of PPT and CPM; 53.0% were women in the age range 18–71 years. Participants were instructed to fast at least 6 h before testing. Demographic information and questionnaire responses were collected prior to testing, and data on the use of pain medications, including both prescriptions and over‐the‐counter drugs, were recorded on the testing day. A total of 1269 (57.7%) participants from the pain substudy of the baseline cohort participated in the pain substudy of the 5‐year follow‐up investigation; 52.0% were women and age ranged from 24 to 76 years.

### 2.2 Ethical Statement

The authors assert that all procedures contributing to this work comply with the ethical standards of the relevant national and institutional committees on human experimentation and with the Helsinki Declaration (World Medical Association 2013). All procedures involving human subjects/patients were approved by the Ethical Committee of the Capital Region (H‐3‐2012‐015), and all participants gave written informed consent.

### 2.3 Dependent Variables

#### 2.3.1 Functional Somatic Disorder

The unifying diagnostic construct of Bodily Distress Syndrome was used as operationalisation of FSD. It divides patients into two subgroups: a single‐organ subgroup (i.e., individuals with symptoms from one or two of four symptom clusters: cardiopulmonary, gastrointestinal, musculoskeletal and general symptoms/fatigue) and a multi‐organ subgroup (i.e., individuals with symptoms from at least three of the four symptom clusters) (Fink et al. 2007). Cases with FSD (including both the single‐ and multi‐organ type) were identified by the self‐reported Bodily Distress Syndrome Checklist (Budtz‐Lilly et al. 2015) assessing physical symptoms which had been bothering within the last 12 months.

In this paper, the FSD diagnosis is conceptualised as Bodily Distress Syndrome, but we also include analyses on three well‐known functional somatic syndromes: Irritable bowel (IB), chronic widespread pain (CWP) and chronic fatigue (CF). Individuals with these conditions were identified with self‐reported validated symptom questionnaires including bothersome symptoms within the last 12 months. IB was defined according to the criteria by Kay and Jorgensen (1996), CWP was defined according to the American College of Rheumatology Criteria (Wolfe et al. 2010) and the definition by White et al. (1999) and CF was defined according to the criteria by Chalder et al. (1993).

Newly developed/incident cases constituted individuals without FSD, IB, CWP or CF, respectively, at baseline but with FSD, IB, CWP or CF at follow‐up. The reference group constituted individuals with no FSD, IB, CWP or CF at either baseline or follow‐up.

### 2.4 Independent Variables

#### 2.4.1 Pressure Pain Threshold

Pressure pain threshold (PPT) was measured at both baseline and follow‐up using a pressure algometer (SBMEDIC Type II; Somedic Senselab AB). Measurements were taken at two sites: over the tibialis anterior muscle, located 10 cm distal to the apex of the patella on the non‐dominant side and over the upper trapezius muscle, positioned 10 cm from the acromion in a direct line with the neck on the non‐dominant side. For both sites, the final PPT value was calculated as the mean of three consecutive recordings.

#### 2.4.2 Conditioned Pain Modulation

Conditioned pain modulation (CPM) was assessed exclusively at baseline using cold pressure stimulation. Following the initial PPT measurement over the tibialis anterior muscle at baseline, participants immersed their non‐dominant hand in a circulating water bath maintained at a maximum temperature of 3°C for 2 min. PPTs were then remeasured over the tibialis anterior muscle during the cold pressor test. The final PPT value was determined as the mean of three consecutive recordings with 20 s between each assessment. Changes in PPTs before and after the cold pressor stimulation were interpreted as indicative of the CPM effect at baseline. The CPM Absolute constituted the measure of baseline minus during conditioning, and the CPM Relative constituted the measure of baseline divided by during conditioning.

### 2.5 Covariates

Sex (female/male) and use of pain medication (yes/no) were obtained with dichotomous items at baseline. Age and mental distress were measured as continuous measures at baseline. Eight items from the Symptom‐Check‐List‐90‐R (SCL‐90‐R) (Derogatis 1992) were used as measures of emotional distress including (SCL‐8) (Fink et al. 2004). Sleep quality was assessed by two questions at baseline: ‘How often do you have difficulty falling asleep?’ and ‘How often do you wake up too early?’, both with four answer categories (once a month or more seldom; two to four times a month; one or more times a week; daily). Sleep quality was then categorised into low (one or more times a week or daily for both questions), average (one or more times a week or daily for one of the questions) and high (once a month or more seldom or two to four times per month for both questions).

### 2.6 Statistical Analysis

Statistical analyses were performed using Stata version 18.0 for Windows (StataCorp 2023). Descriptive statistics were presented as medians and interquartile ranges (IQR). For categorical variables, frequencies with percentages were shown. Responders and non‐responders for the baseline pain substudy and the 5‐year follow‐up investigation pain substudy were compared on several baseline parameters with Wilcoxon Rank‐sum tests (continuous variables) and Pearson's chi‐squared tests (categorical variables).

A number of logistic regression analyses were applied including FSD, IB, CWP and CF, respectively, as the primary dependent variable and pain measures (PPT and CPM) as primary independent variables. For all dependent variables, crude analyses were conducted (Model 1) as were analyses controlling for baseline status of sex, age and use of pain medication (Model 2). For analyses on FSD, additional analyses were conducted, also controlling for mental distress and sleep quality at baseline (Model 3). This additional adjustment for mental distress and sleep quality could only be conducted for the FSD group due to a too low number of incident IB, CWP and CF cases. As the CPM effect is relative to the baseline PPT value, all CPM analyses were also controlled for baseline levels of PPT.

Initially, the primary (continuous) independent variables were modelled using restricted cubic splines with five knots at the 5th, 27.5th, 50th, 72.5th and 95th percentiles according to the recommendations by Harrell (2015) to avoid the assumption of a linear effect on the log odds of the outcome. We then tested whether there were any deviations from linearity using a *χ* ^2^‐test (*p* < 0.05) (results not shown). These analyses showed that the assumption of a linear relationship between the primary independent variable and the primary dependent variable was fulfilled for all groups.

Associations were shown as odds ratios (ORs) with 95% confidence intervals (CIs) comparing participants who differed 100 kPa on the PPT measures, 50 kPa on the CPM absolute measure (baseline minus during conditioning) and 20% on the CPM relative measure (baseline divided by during conditioning). These units constituted a priori defined units, pragmatically established with the aim of obtaining a true difference in pain measures between cases and controls if such difference existed. Hence, the units were based on descriptive data as well as clinical experience and had been used in a previous study from the same author group (Petersen et al. 2021).

We further used a dichotomised version of the CPM relative, where an increase in PPT below 20% (coded as 1) after a cold stimulus was considered pathological and an increase in PPT above 20% was considered normal (coded as 0).

## 3 Results

### 3.1 Sample Characteristics

Respondents and non‐respondents of the pain substudy did not differ on baseline measures (Supporting Information, Table S1). Non‐responders for the 5‐year investigation pain substudy were younger and had lower median PPT measured at the Trapezius muscle than responders, but otherwise the two groups did not differ on baseline measures (Table S2).

At the 5‐year follow‐up investigation, 115 participants had developed FSD, 31 had developed IB, 60 had developed CWP and 47 had developed CF. The median age of the included participants was 55 years (IQR: 47–63), and 659 (51.9%) were women (Table 1). At a glance, participants with newly developed FSD, IB, CWP or CF more frequently used pain medication and had low sleep quality compared to the reference group (Table 1). Furthermore, the PPT seemed lower in participants with FSD, IB, CWP or CF, while the CPM was more scattered (Table 1).

**TABLE 1:** Baseline characteristics of newly developed (incident) cases at follow‐up and references (*n* = 1269).

	Reference (*n* = 859)	FSD (*n* = 115)	IB (*n* = 31)	CWP (*n* = 60)	CF (*n* = 47)
Age; median (IQR)	55 (47–63)	54 (47–64)	48 (37–48)	57 (52–66)	49 (39–62)
Female; *n* (%)	416 (48.4)	70 (60.9)	27 (87.0)	35 (58.3)	28 (59.6)
Sleep quality, *n* (%)					
Low	75 (8.7)	27 (23.5)	9 (29.0)	14 (23.3)	12 (25.5)
Average	214 (24.9)	28 (24.4)	10 (32.3)	21 (35.0)	9 (19.2)
High	559 (65.1)	60 (52.2)	12 (38.7)	25 (41.7)	26 (55.3)
Mental distress, SCL‐8; median (IQR)	1 (0–2)	2 (1–5)	4 (2–8)	2 (1–5.5)	2.5 (1–7)
Pain medication; *n* (%)	10 (1.2)	7 (6.1)	2 (6.5)	3 (5.0)	2 (4.3)
VAS‐score; median (IQR)	7 (5–8)	6 (5–8)	8 (6–8)	7 (6–8)	7 (5–8)
PPT tibialis, kPa; median (IQR)	540.3 (386–722.3)	474.3 (345–605.3)	404.7 (290.7–621.3)	455.8 (302.3–651.8)	539.3 (318.7–695.7)
PPT trapezius, kPa; median (IQR)	485 (350–626.7)	440.7 (322.7–574)	395 (293–512.3)	416.3 (299–574.7)	414.3 (302.7–602.3)
CPM absolute, kPa; median (IQR)	172.5 (85.8–281.7)	160.7 (69.3–245.3)	108.8 (34.3–231.3)	148.2 (63.7–281.7)	177.3 (126.7–232.3)
CPM relative, kPa; median (IQR)	33.6 (15.4–57.7)	38.4 (12.9–56.6)	32.1 (9.2–50.1)	35.5 (13.3–56.9)	41.6 (19.2–61.1)
CPM change < 20%; *n* (%)	61 (7.1)	7 (6.01)	5 (16.1)	4 (6.7)	1 (2.1)

*Note:* The Reference group constituted individuals without any FSD, IB, CWP or CF at either baseline or follow‐up.

Abbreviations: CF, chronic fatigue; CWP, chronic widespread pain; FSD, functional somatic disorder; IB, irritable bowel.

### 3.2 Pressure Pain Threshold

**FIGURE 2:**
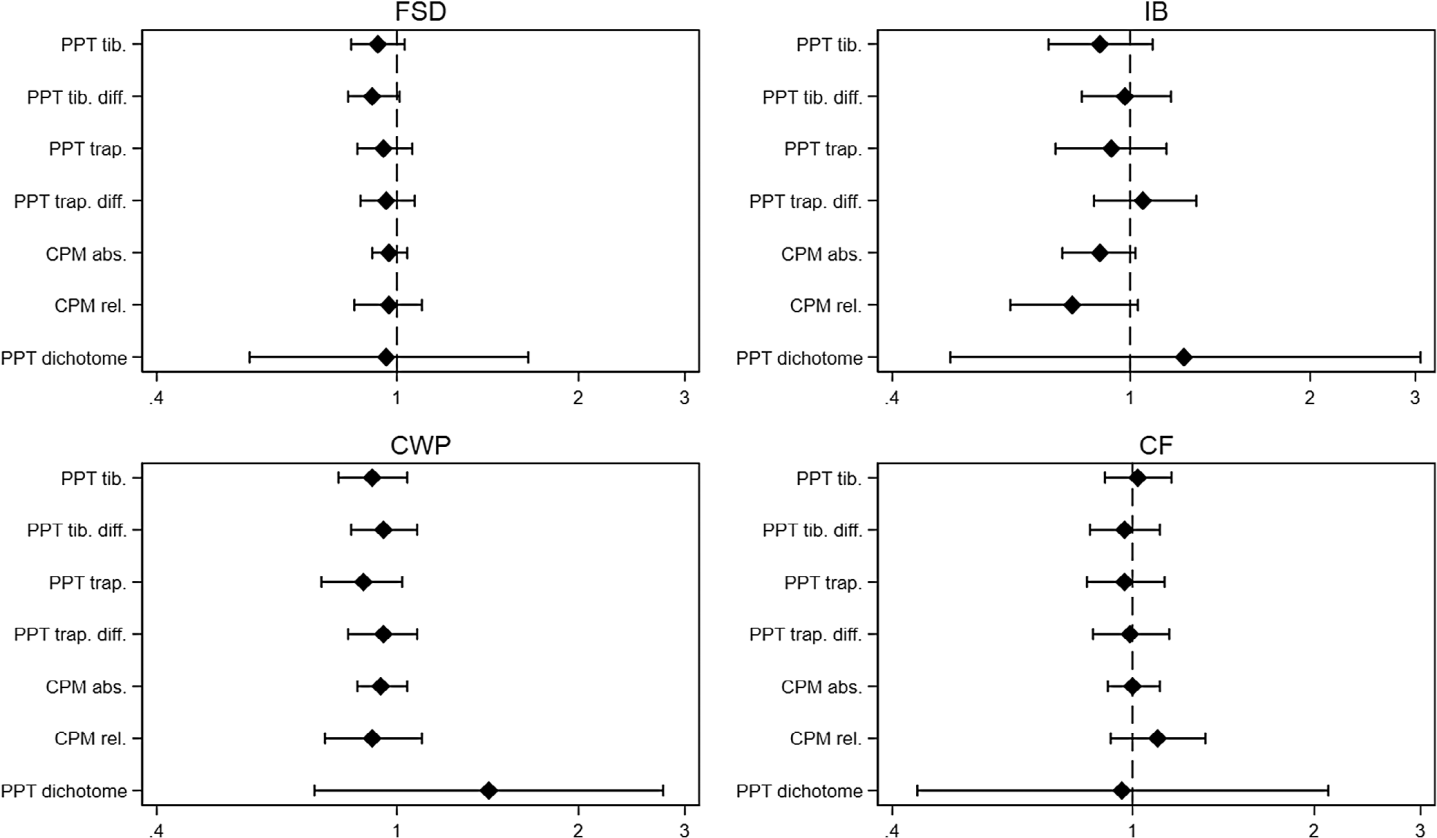
Illustration of odds ratios with 95% confidence intervals for incident functional somatic disorder (FSD), irritable bowel syndrome (IB), chronic widespread pain (CWP) and chronic fatigue (CF). The graph for FSD shows results from the fully adjusted model 3, while the graphs for IB, CWP and CF show results from model 2. CPM, conditioned pain modulation; Diff., difference in levels between baseline and follow‐up; PPT, pressure pain threshold; PPT change < 20%, increase in PPT after the cold stimulus is below 20%.

Besides for some few exceptions in the crude analyses and all the adjusted models, no significant associations were found between baseline PPT levels and the development of FSD, IB, CWP or CF at follow‐up (Table 2 and Figure 2). For example, in the fully adjusted Model 3, comparing participants with a 100‐point difference in baseline PPT measured over the tibialis anterior muscle, the odds of having developed FSD at follow‐up were 0.93 (95% CI: 0.84–1.03).

**TABLE 2:** Pain measures as risk factors for incident FSD. Logistic regression models.

	FSD (*n* = 115)			IB (*n* = 31)		CWP (*n* = 60)		CF (*n* = 47)	
	OR (95% CI)			OR (95% CI)		OR (95% CI)		OR (95% CI)	
	Model 1	Model 2	Model 3	Model 1	Model 2	Model 1	Model 2	Model 1	Model 2
PPT tibialis	**0.89 (0.82–0.98)**	0.92 (0.83–1.01)	0.93 (0.84–1.03)	**0.79 (0.65–0.95)**	0.89 (0.73–1.09)	0.89 (0.79–1.01)	0.91 (0.80–1.04)	0.98 (0.87–1.11)	1.02 (0.90–1.16)
PPT tibialis diff.	0.91 (0.83–1.00)	0.91 (0.83–1.00)	0.91 (0.83–1.01)	0.98 (0.83–1.15)	0.98 (0.83–1.17)	0.95 (0.84–1.07)	0.95 (0.84–1.08)	0.97 (0.85–1.11)	0.97 (0.85–1.11)
PPT trapezius	0.91 (0.82–1.00)	0.93 (0.83–1.03)	0.95 (0.86–1.06)	**0.80 (0.65–0.98)**	0.93 (0.75–1.15)	0.89 (0.77–1.02)	0.88 (0.75–1.02)	0.92 (0.80–1.07)	0.97 (0.84–1.13)
PPT trapezius diff.	0.96 (0.87–1.06)	0.95 (0.86–1.06)	0.96 (0.87–1.07)	1.04 (0.88–1.25)	1.05 (0.87–1.29)	0.95 (0.84–1.08)	0.95 (0.83–1.08)	0.99 (0.86–1.15)	0.99 (0.86–1.15)
CPM absolute	0.96 (0.90–1.03)	0.96 (0.90–1.03)	0.97 (0.91–1.04)	0.88 (0.77–1.00)	0.89 (0.77–1.02)	0.95 (0.87–1.05)	0.94 (0.86–1.04)	1.00 (0.91–1.11)	1.00 (0.91–1.11)
CPM relative	1.02 (0.91–1.15)	0.95 (0.84–1.09)	0.97 (0.85–1.10)	0.91 (0.72–1.15)	0.80 (0.63–1.03)	0.98 (0.81–1.15)	0.91 (0.76–1.10)	1.11 (0.94–1.30)	1.10 (0.92–1.32)
PPT change < 20%	0.92 (0.56–1.51)	1.01 (0.60–1.69)	0.96 (0.57–1.65)	1.06 (0.44–2.52)	1.23 (0.50–3.06)	1.23 (0.65–2.33)	1.42 (0.73–2.76)	0.98 (0.46–2.11)	0.96 (0.44–2.11)

*Note:* Model 1: Unadjusted analyses. Model 2: Analyses on PPT are adjusted for sex, age and use of pain medication at baseline. Analyses on CPM are adjusted for sex, age, PPT and pain medication at baseline. Model 3: Analyses on PPT are adjusted for sex, age, use of pain medication, mental distress and sleep quality at baseline. Analyses on CPM are adjusted for sex, age, PPT, pain medication, mental distress and sleep quality at baseline. The reference group constituted individuals without any FSD, IB, CWP or CF at either baseline or follow‐up. For estimate 0.89 (PPT tibialis in FSD), *p* = 0.017. For estimate 0.79 (PPT tibialis in IB), *p* = 0.011. For estimate 0.80 (PPT trapezius in IB), *p* = 0.028.

Abbreviations: CF, chronic fatigue; CPM, conditioned pain modulation; CWP, chronic widespread pain; Diff., difference in levels between baseline and follow‐up; FSD, functional somatic disorder; IB, irritable bowel; PPT change < 20%, increase in PPT after the cold stimulus is below 20%; PPT, pressure pain threshold.

Additionally, changes in PPT from baseline to follow‐up were not associated with incident FSD (OR = 0.91, 95% CI: 0.83–1.00).

### 3.3 Conditioned Pain Modulation

In both the crude analyses and the adjusted models, no significant associations were found between baseline CPM levels and the development of FSD, IB, CWP or CF (Table 2 and Figure 2). For example, in the fully adjusted Model 3, comparing participants with a 50‐point higher baseline CPM Absolute, the odds of having developed FSD at follow‐up was 0.97 (95% CI: 0.91–1.04).

Additionally, no associations were found between having a PPT change < 20% following cold water stimulation and the risk of developing any of the conditions (Table 2 and Figure 2).

## 4 Discussion

To our knowledge, this is the first large population‐based study examining PPT and CPM as potential risk factors for the development of FSD, IB, CWP and CF. Overall, we did not find any significant associations between PPT and CPM and incident FSD, IB, CWP and CF. This study could therefore not support the hypothesis that altered pain regulation increases the risk of developing these conditions.

This is in contrast to a study by Naugle et al. (2020) where lower CPM (but not PPT) scores predicted a higher likelihood of having prolonged post‐traumatic headache following mild traumatic brain injury at 4 months post‐injury. This was, however, based on a small (*n* = 44) and selected patient group recruited from trauma centres. Thus, publication bias cannot be ruled out.

When interpreting the present findings in relation to the pathophysiology of FSD, it is important to recognise that experimentally assessed pain modulation represents an indirect approximation of endogenous pain regulatory processes. In human research, CPM is commonly used to characterise diffuse noxious inhibitory control‐like phenomena, but it does not provide a direct measure of specific descending pain modulatory pathways (Kennedy et al. 2016; Yarnitsky et al. 2015). The use of CPM has been debated as CPM effects may be influenced by cognitive and contextual factors such as attention and expectancy. In addition, the magnitude of CPM can vary depending on the conditioning stimulus employed, which may affect the classification of individuals as having normal or impaired pain modulation, even when stimulus parameters are comparable. These considerations highlight the variability inherent to CPM paradigms rather than limitations specific to the present study (Albusoda et al. 2018). Alterations in PPT and CPM are often interpreted within a framework of central sensitisation (Arendt‐Nielsen 2015; Bilika et al. 2024; Bourke et al. 2015; den Boer et al. 2019). However, while central sensitisation describes increased responsiveness within the central nervous system, evidence supporting a direct causal role in the development of FSD remains limited. This underscores the complexity of linking experimental pain measures to clinical symptomatology and may not completely explain the lack of observed associations in the present study (Stussman et al. 2018; Vaegter and Graven‐Nielsen 2016).

Regardless of what PPT and CPM measures represent in a pathophysiological perspective, our findings suggest that altered values may either indicate vulnerability to or protection from the development of FSD. Therefore, we regard the observations of altered pain measures to reflect altered pain regulation not in a strict pathophysiological sense but within a biopsychosocial framework. Studies show that chronic pain patients with fibromyalgia and healthy volunteers expressed difficulties and variability in rating experimental pain (Gerhardt et al. 2017; Gil‐Ugidos et al. 2024; Potvin and Marchand 2016; Stussman et al. 2018) and that differences in doctors' and patients' understanding of pain in a biomedical clinical pain research setting exist (Gormsen 2025). Alterations in pain measures like PPT and CPM in FSD may reflect dysregulation influenced by biopsychosocial factors, not only in pain regulation but also in the broader regulation of symptoms. This is due to the interconnectedness of pain processing systems with various other brain and nervous system networks, including the autonomic nervous system, which helps explain the wide range of symptoms such as palpitations, sweating, gastrointestinal issues and fatigue.

Taking these methodological considerations into account, our results do not provide evidence for a direct role of altered pain processing in the development of FSD. Nonetheless, they do not preclude the possibility that disturbances in central processing, not limited to pain systems, may contribute to the diverse symptom manifestations observed in FSD (Loeser and Ballantyne 2024; Löwe et al. 2024).

### 4.1 Strengths and Limitations

The current study has several strengths: First, we included a large, randomly invited sample from the general population (*n* = 2198) with an almost equal distribution of males and females. In addition, we were able to incorporate 5‐year follow‐up data on FSD status for these participants. In contrast, most other studies are based on highly selected patient samples—often predominantly female—and typically lack longitudinal data, which may introduce systematic bias related to somatic or psychological comorbidities, which may be the reason these individuals end up in specialised clinical settings. By using a population‐based study design, the risk of selection bias is minimised, making it possible to generalise the findings to other adult populations. Secondly, given the variety of criteria proposed for identifying FSD (Burton et al. 2020), we incorporated different approaches to define FSD in our study. This was done to better reflect the heterogeneous nature of these conditions, which can present as both mono‐ and multi‐systemic. Thirdly, we applied well‐established and validated symptom questionnaires to establish various definitions of FSD. Finally, the heterogeneity in methodology has been criticised in studies with PPT and CPM. However, this study used methods that show high reliability in both healthy controls and pain patients.

Nonetheless, our study also has some limitations that need to be addressed. First, the definition of FSD groups in our study was based on self‐reported symptoms rather than diagnostic interviews or clinical assessments. This approach may raise concerns regarding the validity of case identification, as it introduces a risk of misclassification bias. It is possible that our sample included individuals with milder symptoms compared to those typically seen in clinical populations, which could help explain some of the negative findings. However, we applied symptom severity cut‐offs, including only symptoms perceived as bothersome when defining FSD cases. Secondly, the response rate of 57.7% for the 5‐year follow‐up pain study may be considered low. While the risk of selection bias is significantly reduced compared to clinical studies, it cannot be entirely ruled out. Third, the number of participants who developed FSD during follow‐up was relatively small. This raises the question of whether statistical power was sufficient. Nevertheless, the relatively narrow confidence intervals for most estimates suggest that the analyses had reasonable power. Furthermore, the relatively small number of participants developing FSD entailed that we had to prioritise which confounders to adjust for. Therefore, it was not possible to adjust for all relevant confounders, for example, physical activity. Lastly, CPM was only assessed at baseline because CPM testing was discontinued due to adverse reactions associated with the cold pressor procedure. As a result, longitudinal changes in endogenous pain modulation could not be examined and the potential contribution of changes in CPM over time to the development of FSD remains unclear.

### 4.2 Conclusion

No significant associations were found between baseline measures of PPT or CPM and the development of FSD, IB, CWP and CF in this population‐based study a with five years follow‐up. However, uncertainty persists due to possible outcome misclassification, a response rate of 57.7% for the 5‐year follow‐up pain substudy, low event numbers and measurement limitations, and call for more research to clarify the underlying mechanisms of FSD.

## Author Contributions

T.J., T.M.D., S.S., T.B.W.C. and M.W.P. contributed to the conception and design of the study. M.W.P. performed the analyses. L.K.G. and M.W.P. interpreted the data and drafted the article. T.J., T.M.D., S.S. and T.B.W.C. contributed to the interpretations of the data. All authors discussed the results and contributed to critically revising the article for important intellectual content. All authors read and approved the final version of the article.

## Funding

This work was supported by grants from the Danish foundations The Lundbeck Foundation (Grant R155‐2013‐14070) and TrygFonden (Grant 7‐11‐0213).

## Conflicts of Interest

The authors declare no conflicts of interest.

## Supporting information


**Table S1:** Baseline characteristics of the baseline pain substudy responders and non‐responders.
**Table S2:** Baseline characteristics of the 5‐year follow‐up pain substudy responders and non‐responders.

## Data Availability

Data are available upon reasonable request to the corresponding author.
