# Supplementary material for: Are Conditioned Pain Modulation and Pain Sensitivity Risk Factors for the Development of Functional Somatic Disorder? A Longitudinal Population‐Based Study
Source: Eur J Pain. 2026 Jul 4;30(6):e70326. doi: 10.1002/ejp.70326 (PMC13332678; doi:10.1002/ejp.70326)
Supplement: Supplementary file 1 — Table S1: Baseline characteristics of the baseline pain substudy responders and non‐responders. Table S2: Baseline characteristics of the 5‐year follow‐up pain substudy responders and non‐responders. [file EJP-30-0-s001.docx]

**Supplemental Materials for the paper**

Are conditioned pain modulation and pain sensitivity risk factors for the development of functional somatic disorder? A longitudinal population-based study

|  | **Responders (n=2,198)** | **Non-responders (n=5,295)** |
| --- | --- | --- |
| **Age;**  **median (IQR)** | 53 (43-62) | 54 (44-64) |
| **Female; n (%)** | 1,164 (53.0) | 2,873 (54.3) |
| **Sleep quality; n (%)** |  |  |
| Low | 297 (13.5) | 765 (14.5) |
| Average | 587 (27.2) | 1,489 (28.1) |
| High | 1,298 (58.3) | 3,010 (56.9) |
| **Mental distress, SCL-8; median (IQR)** | 1 (0-3) | 1 (0-4) |
| **Pain medication; n (%)** | 56 (2.6) | 175 (3.3) |
| **VAS-score; median (IQR)** | 7 (5-8) | NA |
| **PPT Tibialis, kPa; median (IQR)** | 551.7 (365-694) | NA |
| **PPT Trapezius, kPa; median (IQR)** | 446 (326-601) | NA |
| **CPM Absolute, kPa; median (IQR)** | 177.7 (91-281) | NA |
| **CPM Relative, kPa; median (IQR)** | 36.0 (17.0-60.2) | NA |
| **CPM change < 20%; n (%)** | 157 (7.14) | NA |

**Table S1: Baseline characteristics of the baseline pain substudy responders and non-responders.**

Abbreviations: IQR = interquartile range; SCL-8 = Symptom checklist for mental distress; VAS = Visual Analogue Scale;; PPT = Pressure pain threshold; CPM = Conditioned pain modulation; kPa = Kilopascal; NA = not applicable as these data were not collected for non-responders.

|  | **Responders (n=1,269)** | **Non-responders (n=929)** |
| --- | --- | --- |
| **Age; median (IQR)** | 55 (47-63) | 49 (37-60)* |
| **Female; n (%)** | 659 (51.9) | 505 (54.4) |
| **Sleep quality; n (%)** |  |  |
| Low | 164 (12.9) | 133 (14.3) |
| Average | 343 (27.0) | 254 (27.3) |
| High | 751 (59.2) | 530 (57.1) |
| **Mental distress, SCL-8; median (IQR)** | 1 (0-3) | 1 (0-4) |
| **Pain medication; n (%)** | 31 (2.4) | 25 (2.7) |
| **VAS-score; median (IQR)** | 7 (5-8) | 7 (5-8) |
| **PPT Tibialis, kPa; median (IQR)** | 518.7 (370-697.3) | 503 (360.7-683) |
| **PPT Trapezius, kPa; median (IQR)** | 461.3 (332.3-618) | 424.3 (312.7-585.7)* |
| **CPM Absolute, kPa; median (IQR)** | 171.3 (87-280) | 187.5 (99.2-282.3) |
| **CPM Relative, kPa; median (IQR)** | 34.7 (15.6-59.0) | 37.6 (18.8-61.9) |
| **CPM change < 20%; n (%)** | 87 (6.9) | 70 (7.5) |

**Table S2: Baseline characteristics of the 5-year follow-up pain substudy responders and non-responders.**

* Indicates significant difference (Wilcoxon Rank-sum test).

Abbreviations: IQR = interquartile range; SCL-8 = Symptom checklist for mental distress; VAS = Visual Analogue Scale;; PPT = Pressure pain threshold; CPM = Conditioned pain modulation; kPa = Kilopascal.
